# antiSMASH 6.0: improving cluster detection and comparison capabilities

**DOI:** 10.1093/nar/gkab335

**Published:** 2021-05-12

**Authors:** Kai Blin, Simon Shaw, Alexander M Kloosterman, Zach Charlop-Powers, Gilles P van Wezel, Marnix H Medema, Tilmann Weber

**Affiliations:** The Novo Nordisk Foundation Center for Biosustainability, Technical University of Denmark, Kgs. Lyngby, Denmark; The Novo Nordisk Foundation Center for Biosustainability, Technical University of Denmark, Kgs. Lyngby, Denmark; Institute of Biology, Leiden University, Leiden, The Netherlands; Bioinformatics, Lodo Therapeutics, New York, USA; Institute of Biology, Leiden University, Leiden, The Netherlands; Netherlands Institute of Ecology (NIOO-KNAW), Wageningen, The Netherlands; Institute of Biology, Leiden University, Leiden, The Netherlands; Bioinformatics Group, Wageningen University, Wageningen, The Netherlands; The Novo Nordisk Foundation Center for Biosustainability, Technical University of Denmark, Kgs. Lyngby, Denmark

## Abstract

Many microorganisms produce natural products that form the basis of antimicrobials, antivirals, and other drugs. Genome mining is routinely used to complement screening-based workflows to discover novel natural products. Since 2011, the "antibiotics and secondary metabolite analysis shell—antiSMASH" (https://antismash.secondarymetabolites.org/) has supported researchers in their microbial genome mining tasks, both as a free-to-use web server and as a standalone tool under an OSI-approved open-source license. It is currently the most widely used tool for detecting and characterising biosynthetic gene clusters (BGCs) in bacteria and fungi. Here, we present the updated version 6 of antiSMASH. antiSMASH 6 increases the number of supported cluster types from 58 to 71, displays the modular structure of multi-modular BGCs, adds a new BGC comparison algorithm, allows for the integration of results from other prediction tools, and more effectively detects tailoring enzymes in RiPP clusters.

## Graphical Abstract

**Graphical Abstract:**
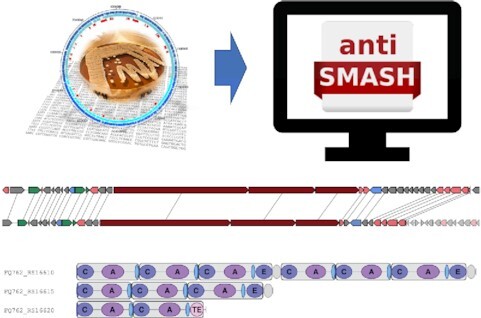
Here, we present version 6 of the secondary/specialized metabolite genome mining platform antiSMASH with improved detection capabilities, a new cluster compare feature and many further improvements.

## INTRODUCTION

Natural compounds produced by microorganisms form the basis of many drugs (1). Traditionally, new compounds were discovered by extracting, chemically isolating, purifying, and then testing from natural sources. This approach can now be complemented by sequencing and subsequent mining of genome and metagenome data to identify natural product biosynthetic pathways (2). Software tools to assist researchers in their natural product genome mining tasks have existed for over a decade. Recently published or updated examples include BAGEL (3), PRISM (4), RiPPER (5) and TOUCAN (6). For more in-depth reviews on the topic, see (7–10).

Since its initial release in 2011, antiSMASH (11–15) has become the most widely used tool for mining microbial genomes for secondary/specialised metabolite (SM) biosynthetic gene clusters (BGCs) and is regarded as the gold standard. An ecosystem of independent tools incorporating or utilising antiSMASH results has developed over the years, such as the antibiotics resistance target seeker ARTS (16), the mass-spectronomy-guided peptide mining tool Pep2Path (17), the sgRNA design tool CRISPY-web (18), the BGC classification and clustering platform BiG-SCAPE (19), or its big data BGC clustering cousin BiG-SLiCE (20). antiSMASH is also used to annotate BGCs in many genomic as well as BGC-oriented databases, such as the Joint Genome Institute's Integrated Microbial Genomes database with its Atlas of Biosynthetic gene Clusters IMG-ABC (21), the MicroScope platform for microbial genome annotation and analysis (22), the MIBiG database of manually curated BGCs (23), the BGC family database BiG-FAM (24), and, of course, the antiSMASH database (25).

**Figure 1.**
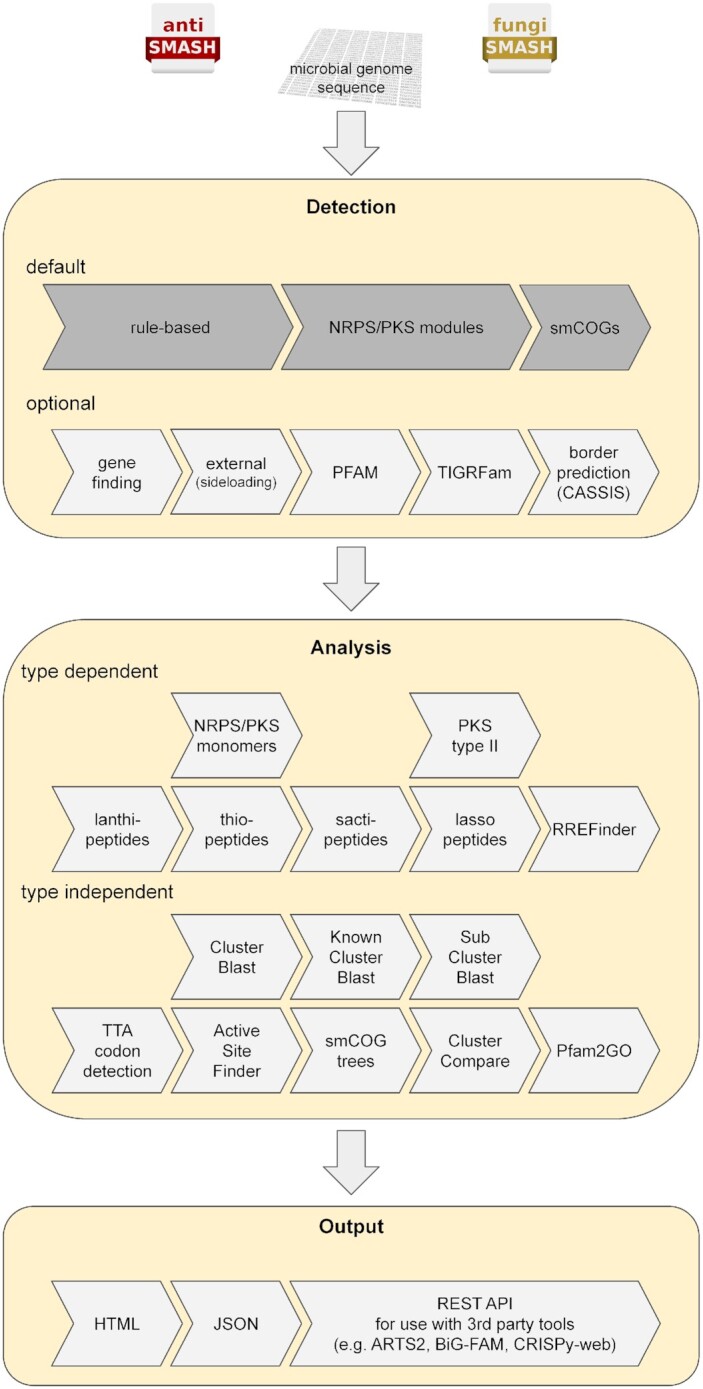
Schematic workflow of the antiSMASH secondary/specialized metabolite genome mining platform.

antiSMASH uses a rule-based approach to identify many different types of biosynthetic pathways involved in SM production. More in-depth analyses are performed for BGCs encoding non-ribosomal peptide synthetases (NRPSs), type I and type II polyketide synthases (PKSs), lanthipeptides, lasso peptides, sactipeptides, and thiopeptides, for which cluster-specific analyses can provide more information about the biosynthetic steps performed and thus also provide more detailed predictions on the compound(s) produced (Figure 1).

Here we present version 6 of antiSMASH. It extends and improves upon previous versions by adding and improving BGC detection rules, making the modular structure of multimodular enzymes more accessible, introducing an additional, more robust, cluster comparison tool, and improving the interoperability with other gene cluster annotation tools.

## NEW FEATURES AND UPDATES

### New cluster types

antiSMASH uses manually curated and validated “rules" that define which core biosynthetic functions need to exist in a genomic region in order to constitute a BGC. To identify these biosynthetic functions, antiSMASH uses profile hidden Markov models (pHMMs) from PFAM (26), TIGRFAMs (27), SMART (28), BAGEL (3), Yadav *et al.* (29), or custom models. antiSMASH 5 contained rules for 58 different BGC types (15), version 6 increases this number to 71. Especially in focus for this release were the rules for ribosomally synthesized and post-translationally modified peptides (RiPPs). The lanthipeptide rule was split up into individual rules for classes I through IV. Several RiPP families that were previously jointly designated as “bacteriocins" are now identified by specific rules. The term “bacteriocins" was therefore deprecated and replaced by “RiPP-like", which is defined by profiles that are frequently associated with RiPPs but are insufficient to detect RiPP clusters by themselves. The old “head_to_tail" rule was folded into the “sactipeptide" rule because they covered the same class of RiPPs. New rules were added for class V lanthipeptides, lanthidines, thioamitides, ranthipeptides, PQQ- and mycofactocin-like redox cofactors, epipeptides, cyclic lactone autoinducers, and spliceotides. Outside of RiPPs, antiSMASH added support for BGCs producing thioamide-containing non-ribosomal peptides, tropodithietic acid, *Serratia*-type prodigiosins, non-alpha poly-amino acids and pyrrolidines.

### Module detection

**Figure 2.**
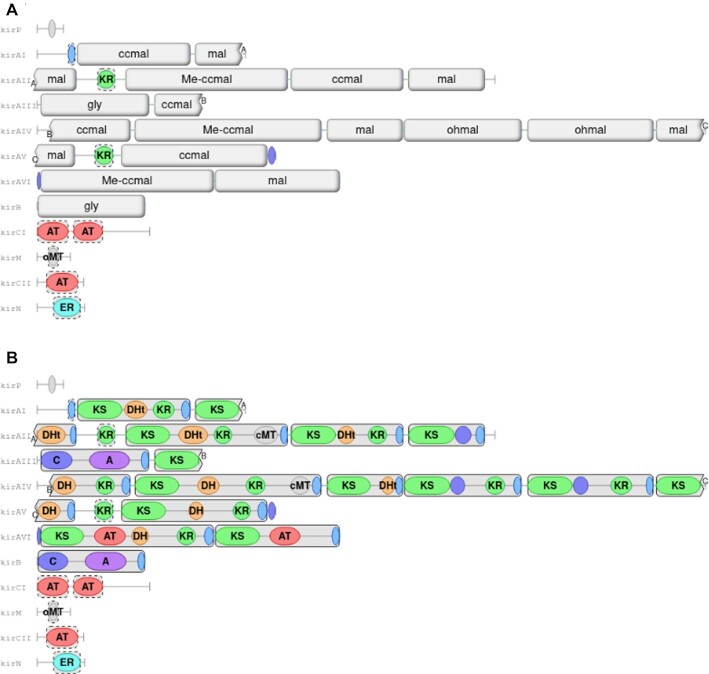
The NRPS/PKS domain view of the kirromycin biosynthetic gene cluster (NCBI ID: AM746336.1), consisting of trans-AT PKS, modular type I PKS and NRPS modules. **A** View with module lids, displaying the monomer predicted to be integrated into the final product. The jagged module edges on KirAI/AII/AIII/AIV show modules that are split across different protein-coding sequences, with the small lettering next to the edges indicating how the modules link up. **B** View with the module lids hidden, revealing the underlying protein domains.

Nonribosomal peptide synthetases, non-iterative type I polyketide synthases and trans-AT polyketide synthases are large, multimodular enzymes (for a review, see (30)). While antiSMASH has always detected the individual enzymatic domains in these megaenzymes, it now also detects and displays the modules explicitly (Figure 2A). Module detection allows for better prediction of modifications made to the respective monomers during biosynthesis. Hence, displaying the modules makes it easier for researchers to interpret the likely biosynthetic mechanisms of an enzymatic assembly line encoded by a BGC. In order to examine the protein domains making up each module, hovering the mouse cursor over a module will send the module lid to the background and will reveal the domains. For a more complete view of all protein domains, module monomer display can be turned off using the “show module domains" toggle button above the graph to hide all module lids (Figure 2B).

### ClusterCompare

**Figure 3.**
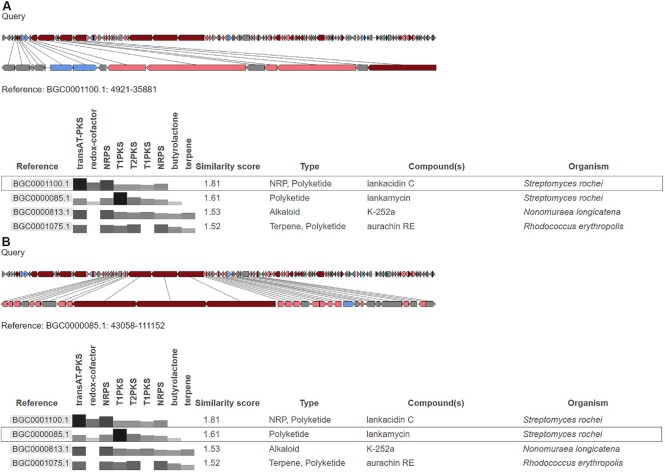
ClusterCompare output for the *Streptomyces rochei* large linear plasmid pSLA2-L (NCBI ID: NC_004808.2), which is densely packed with secondary metabolite biosynthetic genes (see (39)) with the MIBiG dataset in protocluster-to-region mode. Lines connect pairs of protein-coding genes with the highest similarity to make conserved functions more visible even at different scaling of query and reference. The comparisons show the similarity of (**A**) the left part of the region to the trans-AT PKS, NRPS hybrid cluster responsible for lankacidin C production (MIBiG ID: BGC0001100.1), as well as (**B**) the middle part of the region to the modular PKS type I cluster responsible for lankamycin biosynthesis (MIBiG ID: BGC0000085.1). The example illustrates how ClusterCompare can be used to distinguish between hybrid gene clusters and adjacent gene clusters that are part of the same region, based on comparison with individual reference BGCs.

Since the release of antiSMASH 1, antiSMASH has provided a comparison of the identified region to similar clusters in other genomes via ClusterBlast (see (11) for a description of the algorithm). As the ClusterBlast algorithm is based on protein sequence comparisons by local alignments (initially using BLAST (31), now using DIAMOND (32)), it does not perform optimally on multimodular enzymes like NRPSes and PKSes. Of particular note, BGCs with very different module counts can score similarly if a single module has a good match. To address this issue, we have added a new ClusterCompare algorithm (Shaw *et al.*, manuscript in preparation) to antiSMASH 6. Like ClusterBlast, ClusterCompare builds on a local protein-alignment-based sequence comparison, but only uses that as one part of the comparison score. Additional parts of the score are the gene synteny and the presence/absence of biosynthetic components of the query and reference gene clusters, based on antiSMASH annotations of each. Biosynthetic components are the collection of gene products matching one of antiSMASH’s BGC detection profiles, gene products with a functional annotation due to either their presence in an antiSMASH detection rule or based on the classification of their secondary metabolite clusters of orthologous groups (smCOG) class, and, if applicable, NRPS and PKS domains. All score parts are scaled to a 0 to 1 range, and the final score for a single comparison is calculated as the geometric mean of the parts. The top hits in the reference database are displayed in a table, along with the scoring information. Selecting a table row will also display a pairwise comparison of the query and reference clusters (Figure 3).

### Sideloading

antiSMASH exists in an ecosystem of BGC prediction and analysis tools. While tools like ARTS (16) and BiG-SCAPE (19) sit downstream of antiSMASH and consume antiSMASH results, other tools like ClusterFinder (33) and DeepBGC (34) offer alternative, machine-learning based methods to predict gene clusters and thus function more in parallel to antiSMASH’s rule based cluster detection. Depending on the research question and the desired sensitivity vs. specificity trade-off, there can be a value in preferring one cluster detection over the other (see (8,35) for a discussion). It is not feasible from a development resource perspective to include and maintain all different cluster detection approaches within the antiSMASH pipeline. Hence, to use analysis modules such as ClusterBlast, ClusterCompare, their respective MIBiG-based variants, or PFAM/TIGRFAMs analysis on the identified BGCs regardless of their mode of detection, we have created a JSON-based file format that can be used by external tools to annotate additional regions and clusters in the input and allow antiSMASH to analyse those areas exactly as per the natively predicted regions and clusters. An explanation of the external annotation file format and the currently implemented sideloading functionality is available at https://docs.antismash.secondarymetabolites.org/sideloading/.

### Improved annotation for RiPP clusters

For lanthipeptide, lasso peptide, sactipeptide, and thiopeptide BGCs, antiSMASH 5 already provided more detailed product predictions by detecting the cluster's prepeptide and commonly occurring tailoring enzymes. Because some of the tailoring enzymes can match relatively generic functional profiles, it was not always trivial to determine whether a given enzyme was indeed interacting with the RiPP precursor peptide as a tailoring enzyme or whether it was just an unrelated enzyme that happened to be encoded in the vicinity. Often, RiPP tailoring enzymes will harbour RiPP recognition element (RRE) domains that can identify and bind the RiPP precursor peptide. RRE-Finder (36), which will annotate these RRE domains, has been integrated into antiSMASH 6, thus helping to more confidently identify tailoring enzymes in antiSMASH-detected RiPP clusters. Additionally, an “RRE-containing" detection rule using the RRE-Finder pHMMs was added to the “relaxed" strictness ruleset, allowing antiSMASH to identify potentially novel RiPP clusters for which antiSMASH does not have a specific detection rule set up, as long as that gene cluster contains an RRE.

### Other optimizations

The sequence data used in antiSMASH’s ClusterBlast are based on the records contained in the antiSMASH database. As the antiSMASH database was recently updated to version 3 (25), the ClusterBlast dataset was also refreshed to include 147 517 high quality BGC regions from 388 archaeal, 25 236 bacterial and 177 fungal genomes. The antiSMASH database version 3 is the first version to also contain both archaeal and fungal sequences along with bacterial sequences, so ClusterBlast will now also give more relevant hits for users running antiSMASH on inputs originating from those taxa.

In addition to the region PFAM analysis added in version 5, antiSMASH 6 can now also scan regions using profiles from the TIGRFAMS database (27).

## CONCLUSIONS AND FUTURE PERSPECTIVES

Genome mining with tools like antiSMASH has become an established part of many natural product discovery workflows. With the updates and additions to the feature set, antiSMASH is positioning itself to remain the go-to tool for microbial genome mining for natural products. By improving the interoperability with other tools, the open-source software antiSMASH integrates even better with the thriving ecosystem of computational tools in the natural products field. Future updates will further improve the predictions of the chemical structures of the compounds produced by the detected BGCs. This will help to connect gene clusters to molecules identified via metabolomics or other analytical chemistry approaches (37), and to link up with databases such as GNPS (38).

## DATA AVAILABILITY

The bacteria and fungal versions of antiSMASH 6 can be freely accessed at https://antismash.secondarymetabolites.org and https://fungismash.secondarymetabolites.org, respectively.

The antiSMASH documentation is available at https://docs.antismash.secondarymetabolites.org/.

The antiSMASH source code, licensed under the GNU Affero General Public License (AGPL) v3.0, is available at https://github.com/antismash/antismash. antiSMASH is also available via Docker.
